# Synthesis, structures and Hirshfeld surface analyses of 2-hy­droxy-*N*′-methyl­acetohydrazide and 2-hy­droxy-*N*-methyl­acetohydrazide

**DOI:** 10.1107/S2056989024009526

**Published:** 2024-10-15

**Authors:** Oleksandr V. Vashchenko, Dmytro M. Khomenko, Viktoriya V. Dyakonenko, Rostyslav D. Lampeka

**Affiliations:** a Department of Chemistry, Taras Shevchenko National University of Kyiv, Volodymyrska str. 64/13, 01601 Kyiv, Ukraine; b Enamine Ltd. (www.enamine.net), Winston Churchill str. 78, 02094 Kyiv, Ukraine; c SSI "Institute for Single Crystals" of the National Academy of Sciences of Ukraine, Nauki Ave 60, Kharkiv 61001, Ukraine; d V I Vernadskii Institute of General and Inorganic Chemistry of the National Academy of Sciences of Ukraine, Prospect Palladina 32/34 03680 Kyiv Ukraine; Universität Greifswald, Germany

**Keywords:** crystal structure, hydrazides, methyl­acetohydrazide, regioisomer, Hirshfeld surface analysis

## Abstract

The structures of the title compounds 2-hy­droxy-*N*′-methyl­acetohydrazide, **1**, and 2-hy­droxy-*N*-methyl­acetohydrazide, **2**, both C_3_H_8_N_2_O_2_, as regioisomers differ in the position of the methyl group relative to the N atoms in 2-hy­droxy-acetohydrazide. In the structure of **1**, the 2-hy­droxy-acetohydrazide core [OH—C—C(=O)—NH—NH] is almost planar and the methyl group is rotated relative to this plane. As opposed to **1**, in the structure of **2** all non-hydrogen atoms lie in the same plane. The hydroxyl and carbonyl groups in structures **1** and **2** are in *trans* and *cis* positions, respectively. The methyl amino group and carbonyl group are in the *cis* position relative to the C—N bond in structure **1**, while the amino group and carbonyl group are in the *trans* position relative to the C—N bond in stucture **2.** In the crystal, mol­ecules of **1** are linked by N—H⋯O and O—H⋯N inter­molecular hydrogen bonds, forming layers parallel to the *ab* crystallographic plane. A Hirshfeld surface analysis showed that the H⋯H contacts dominate the crystal packing with a contribution of 55.3%. The contribution of the H⋯O/O⋯H inter­action is somewhat smaller, amounting to 30.8%. In the crystal, as a result of the inter­molecular O—H⋯O hydrogen bonds, mol­ecules of **2** form dimers, which are linked by N—H⋯O hydrogen bonds and a three-dimensional supra­molecular network The major contributors to the Hirshfeld surface are H⋯H (58.5%) and H⋯O/O⋯H contacts (31.7%).

## Abstract 2

The crystal structures of 2-hy­droxy-*N′*-methyl­acetohydrazide and 2-hy­droxy-*N*-methyl­acetohydrazide are reported and discussed.

## 1. Chemical context

*N*-substituted hydrazides are widely used compounds in organic synthesis. Aza-peptides containing the *N*-alkyl hydrazide fragment have been investigated as wide-spectrum anti­biotics (Amabili *et al.*, 2020 ▸), drugs for inflammatory acne treatment (Fournier *et al.*, 2018 ▸), anti­viral agents (Breidenbach *et al.*, 2021 ▸) and selective protease inhibitors (Corrigan *et al.*, 2020 ▸). Additionally, *N*-alkyl hydrazides are very important starting reagents for the synthesis of 1,2-substituted 1,2,4-triazoles (Nguyen & Hong, 2021 ▸; Peese *et al.*, 2020 ▸), 3-substituted 1,3,4-thia­diazol-2-ones and 1,3,4-oxo­diazol-2-ones (Kuzmina *et al.*, 2019 ▸; Bi *et al.*, 2019 ▸), 2,3-di­hydro-1H-pyrazoles (Shaker Ardakani *et al.*, 2021 ▸), and other heterocyclic or spyrocyclic compounds (Kobayashi & Ainai, 2018 ▸; Tian *et al.*, 2022 ▸).

Previously, we have obtained a series of *N*1- and *N*2-alkyl­ated 1,2,4-triazoles (Khomenko *et al.*, 2022 ▸; Ohorodnik *et al.*, 2023 ▸). The separation of the resulting regioisomers was achieved through flash column chromatography. The use of pure *N*-methyl regioisomers of hydrazides in the synthesis of 1,2,4-triazoles allows for the direct formation of the desired *N*1- and *N*2-methyl­ated compounds, thereby eliminating the need for an expensive flash chromatography step.

Usually, the inter­action of carb­oxy­lic acid derivatives with *N*-alkyl hydrazines leads to a mixture of regioisomers (Condon, 1972 ▸), while the desired *N*- or *N′*- regioisomer can be obtained from BOC or CBZ-protected *N*-alkyl hydrazines (Amabili *et al.*, 2020 ▸; Peese *et al.*, 2020 ▸). This method, however, has several disadvantages: expensive reagents, more steps, and the need for protecting other functional groups.
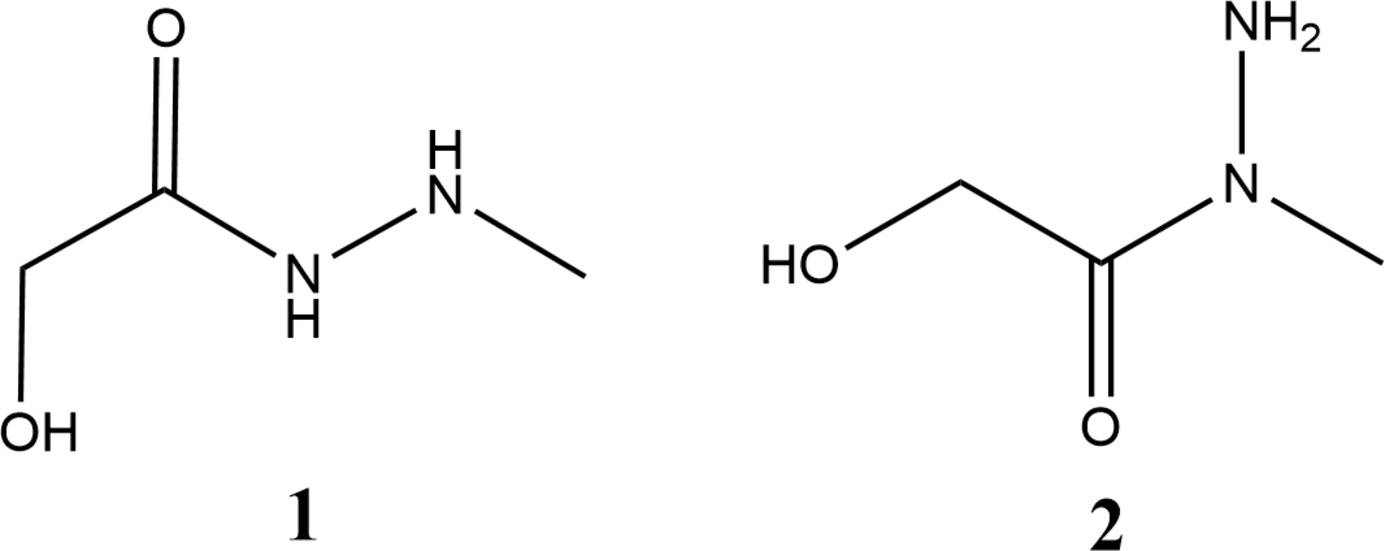


In this work, we report the one-step synthesis and purification procedure of 2-hy­droxy-*N′*-methyl­acetohydrazide (**1**) and 2-hy­droxy-*N*-methyl­acetohydrazide (**2**) using inexpensive reagents, their crystal structures and Hirshfeld surface analyses.

## 2. Structural commentary

**Figure 1:**
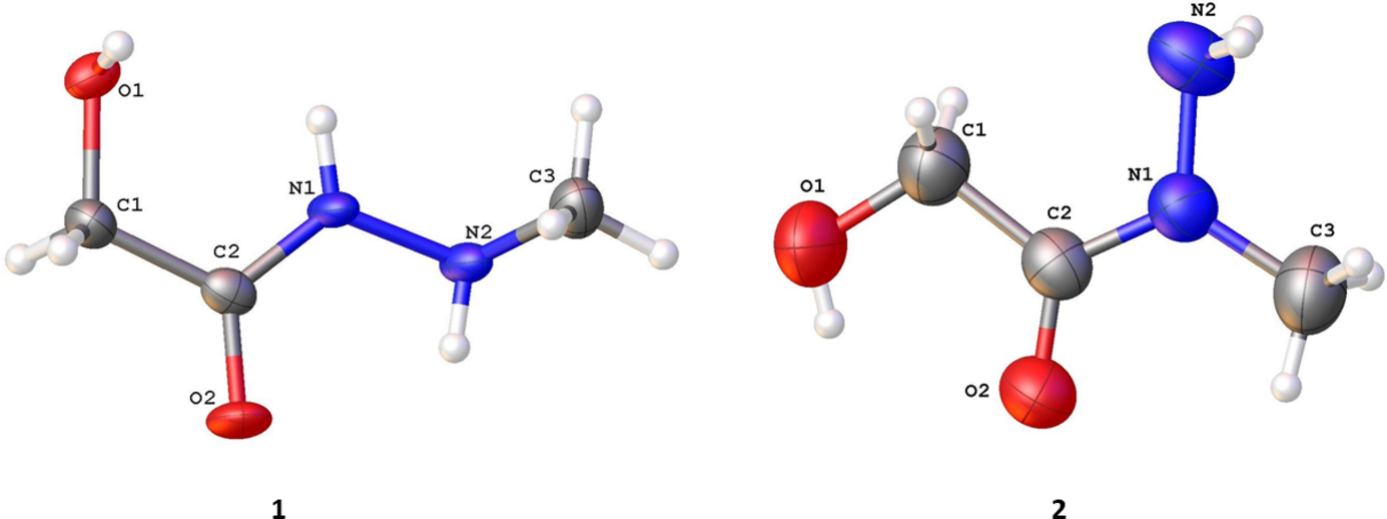
The mol­ecular structures of **1** and **2** with atom labeling and displacement ellipsoids drawn at the 50% probability level.

Structures **1** and **2** are regioisomers and differ in the position of the methyl group relative to the N atoms in 2-hy­droxy-acetohydrazide (Fig. 1 ▸). Compound **1** crystallizes in the ortho­rhom­bic space group *Pbca*, while **2** crystallizes in the monoclinic space group *C*2/*c*.

In the structure of **1**, the 2-hy­droxy-acetohydrazide core [OH—C—C(=O)—NH—NH] is almost planar (r.m.s. deviation is 0.016 Å). The methyl group is rotated relative to this plane [the C2—N1—N2—C3 torsion angle is −124.1 (4)°]. The hydroxyl and carbonyl groups are in *trans* positions. The methyl amino group and carbonyl group are in the *cis* position relative to the C2—N1 bond. The O–C–N–N fragment shows features of conjugation, supported by the pronounced shortening of the C2—N1 [1.300 (6) Å] single bond compared to the average value of 1.355 Å (Orpen *et al.*, 1994 ▸). This may be enhanced by the formation of the N1—H1*A*⋯O2′ inter­molecular hydrogen bond (Table 1 ▸).

As opposed to **1**, in the structure of **2** all non-hydrogen atoms lie in the same plane (r.m.s. deviation is 0.028 Å). The hydroxyl and carbonyl groups are in *cis* positions. The amino group and carbonyl group are in the *trans* position relative to the C2—N1 bond. Both the C2—O2 [1.251 (3) Å] and the N1—N2 [1.434 (3) Å] bonds are elongated compared to the average values of 1.234 and 1.420 Å, respectively (Orpen *et al.*, 1994 ▸). The elongation of the N1—N2 bond, together with the absence of a shortening of the C1—N2 bond, may indicate a slight disruption of the conjugation within the O–C–N–N core. That is consistent with amino group rotation: C2—N1—N2—H torsion angles are +12° and −116°, indicating an in-plane position of the lone pair of the N2 atom, stabilized by the N2—H2*A*⋯O2 and N2—H2*B*⋯O1 inter­molecular hydrogen bonds (Table 2 ▸), so this lone pair cannot participate in the π-conjugation of the O–C–N–N fragment. The minor elongation of the C2=O2 double bond is probably caused by the presence of the inter­molecular bi-directional hydrogen bond O1—H1⋯O2 with the O—H group of an adjacent mol­ecule and the N2—H2*B*⋯O2′′ hydrogen with another mol­ecule (Table 2 ▸).

The N2 atom is pyramidal in both structures **1** and **2** (the sums of the valence angles is 225.93 and 317.93° in **1** and **2**, respectively). The pyramidal configuration of the N2 atom is stabilized by inter­molecular hydrogen bonds O1—H1⋯N2 (in **1**, Table 1 ▸) and N2—H2*B*⋯O1, N2—H2*A*⋯O2 (in **2**, Table 2 ▸).

## 3. Supra­molecular features and Hirshfeld surface analysis

**Figure 2:**
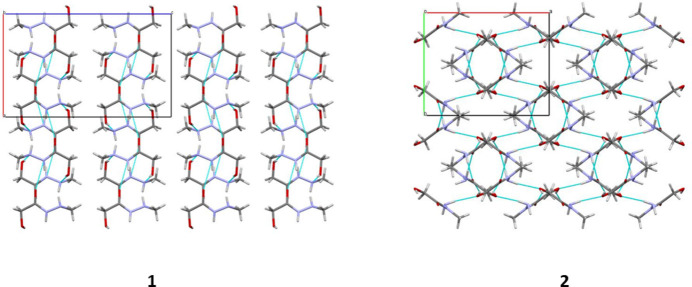
Crystal packing of **1** viewed along the *b* axis (left) and **2** viewed along *c* axis (right). The hydrogen bonds are shown as blue dotted lines.

In the crystal, mol­ecules of **1** are linked by N—H⋯O and O—H⋯N hydrogen bonds (Table 1 ▸), forming layers parallel to the *ab* crystallographic plane (Fig. 2 ▸).

**Figure 3:**
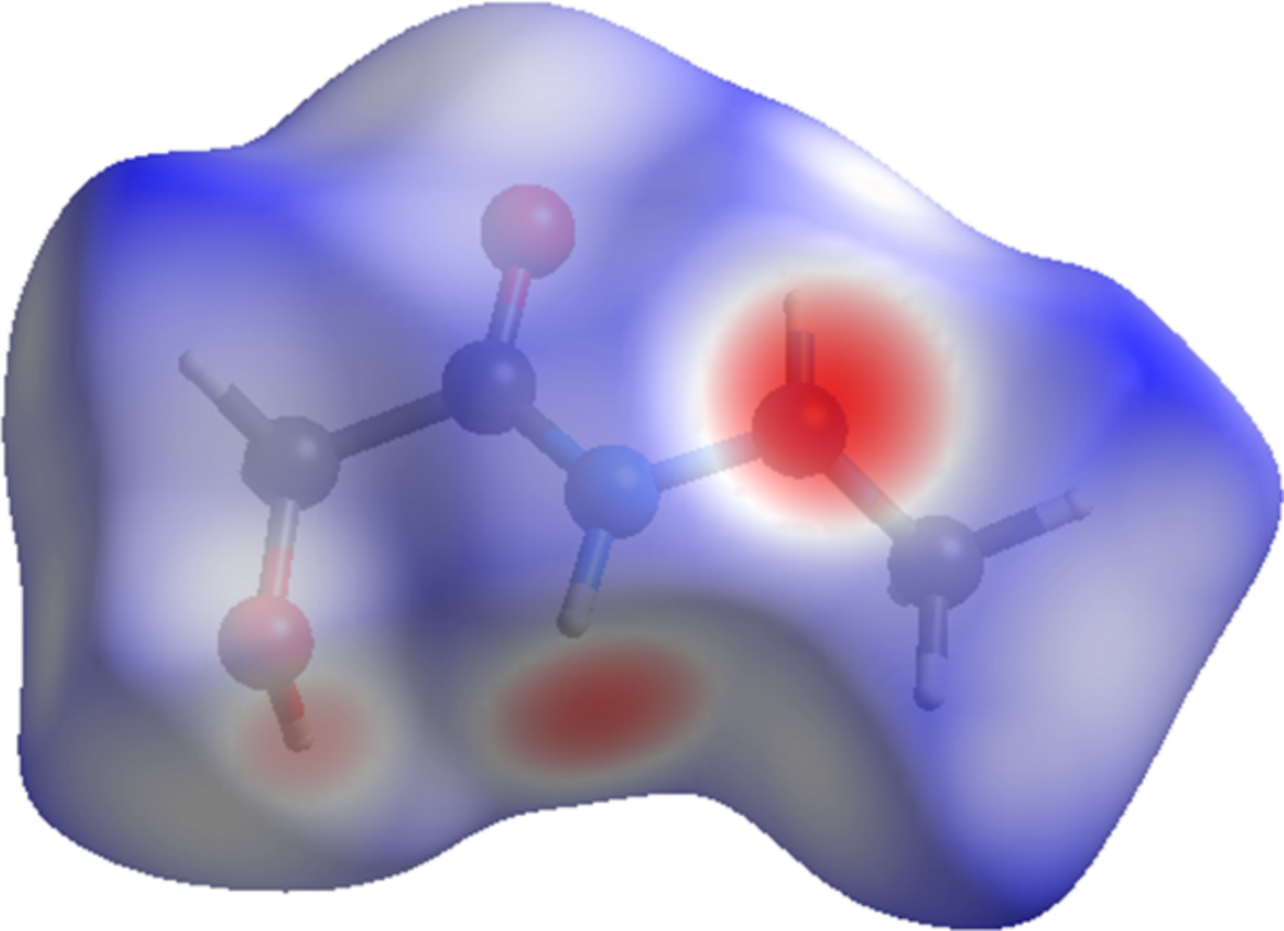
The Hirshfeld surface mapped over *d*_norm_ for visualizing the inter­molecular contacts of compound **1**.

The inter­molecular inter­actions in the crystal structure of **1** were further analyzed by means of the *d*_norm_ property (Fig. 3 ▸) mapped over the Hirshfeld surface (Spackman & Jayatilaka, 2009 ▸), which was calculated using the *CrystalExplorer21* program (Spackman *et al.*, 2021 ▸). The strongest contacts, which are visualized on the Hirshfeld surface as the dark-red spots, correspond to the N—H⋯O and O—H⋯N hydrogen bonds between mol­ecules. The majority of the inter­molecular inter­actions of **1** are weak, and are represented in blue on the Hirshfeld surface.

**Figure 4:**
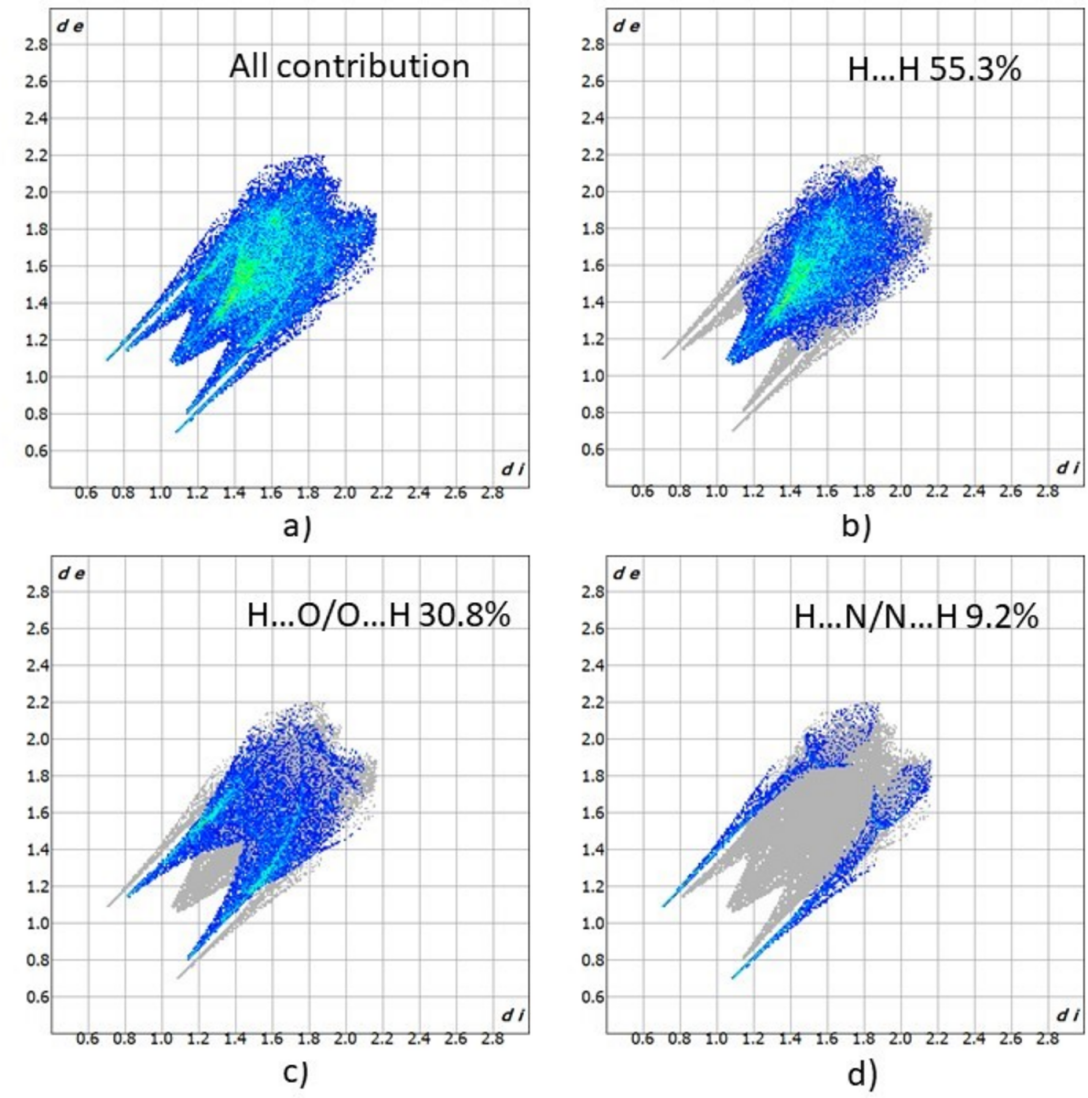
Two-dimensional fingerprint plots for **1** showing (*a*) all inter­actions, and (*b*)–(*d*) delineated into contributions from other contacts (blue areas) [*d*_e_ and *d*_i_ represent the distances from a point on the Hirshfeld surface to the nearest atoms outside (external) and inside (inter­nal) the surface, respectively].

For further exploration of the inter­molecular inter­actions, two-dimensional fingerprint plots (McKinnon *et al.*, 2007 ▸) were generated, as shown in Fig. 4 ▸. The major contributions to the crystal structure are from the H⋯H (55.3%) and H⋯O/O⋯H (30.8%) inter­actions. The N⋯H/H⋯N (9.2%) and O⋯C/C⋯O (2.5%) inter­actions are less impactful in comparison.

In the crystal of **2**, as a result of the O—H⋯O inter­molecular hydrogen bonds (Table 2 ▸) the mol­ecules form dimers, which are linked by N—H⋯O inter­molecular hydrogen bonds to form a 3D supra­molecular network (Fig. 2 ▸).

**Figure 5:**
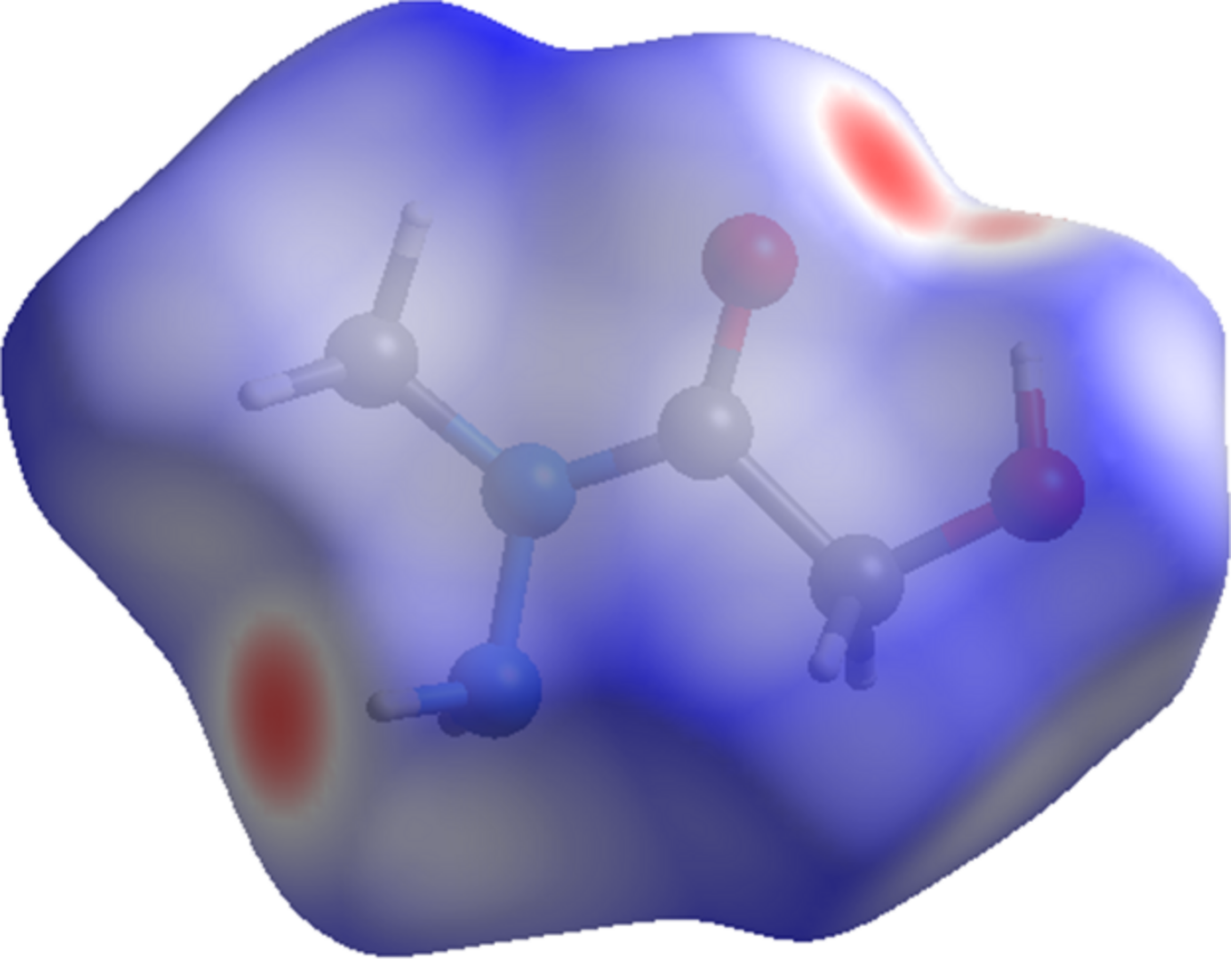
The Hirshfeld surface mapped over *d*_norm_ for visualizing the inter­molecular contacts of compound **2**.

**Figure 6:**
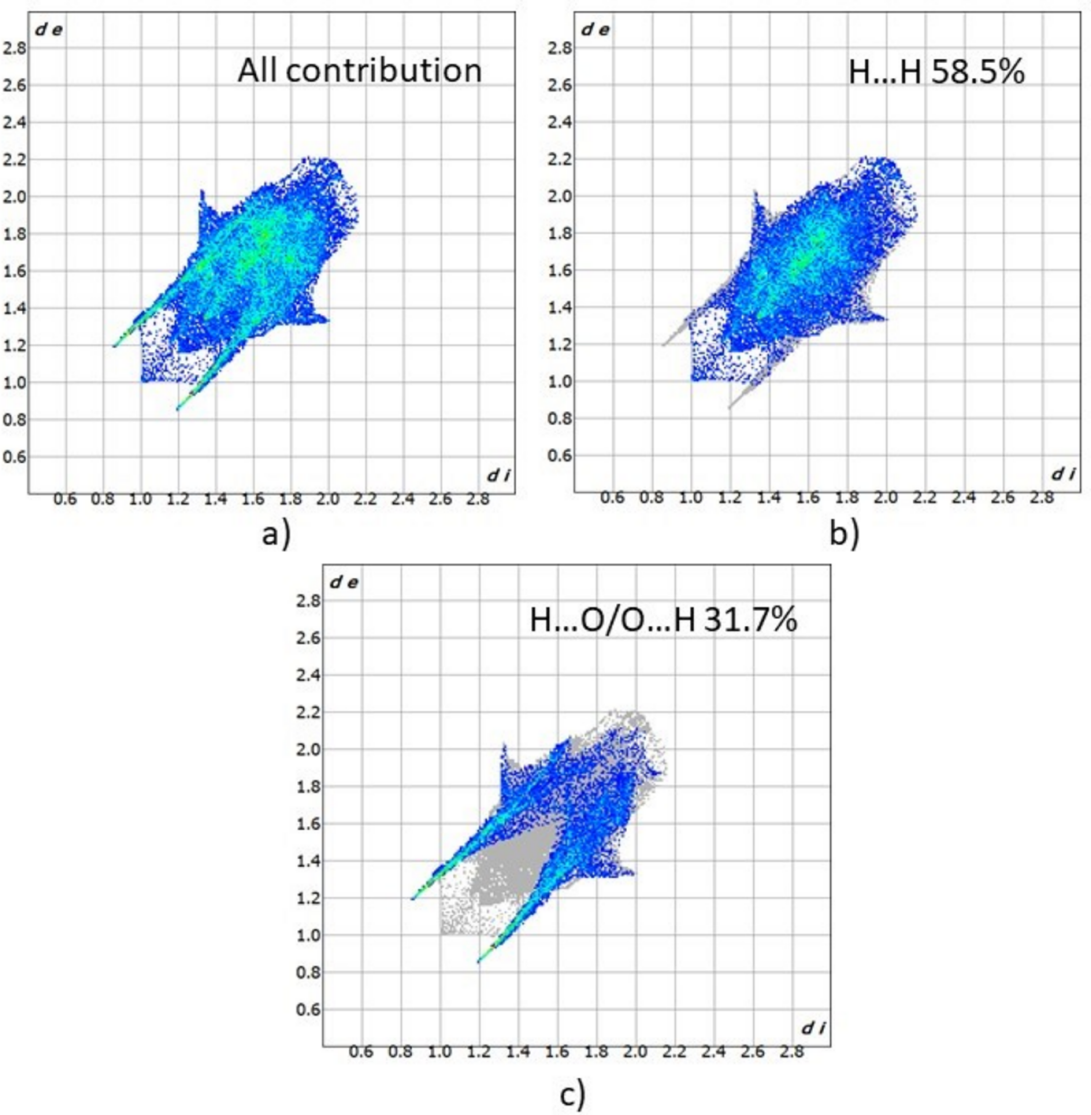
Two-dimensional fingerprint plots for **1** showing (*a*) all inter­actions, and (*b*)–(*c*) delineated into contributions from other contacts (blue areas) [*d*_e_ and *d*_i_ represent the distances from a point on the Hirshfeld surface to the nearest atoms outside (external) and inside (inter­nal) the surface, respectively].

Fig. 5 ▸ shows the Hirshfeld surface of **2** plotted over *d*_norm_ (normalized contact distance) and Fig. 6 ▸ the 2D fingerprint plots. The strongest contacts, which are visualized on the Hirshfeld surface as the dark-red spots, correspond to the O–H⋯O and N—H⋯O hydrogen bonds between mol­ecules. The major contributions to the crystal structure are from the H⋯H(58.5%) and H⋯O/O⋯H (31.7%) inter­action. The N⋯H/H⋯N (4.0%) and H⋯C/C⋯H (3.2%), O⋯N/N⋯O inter­actions are of lower relevance.

## 4. Database survey

A search of the Cambridge Structural Database (CSD, version 2024.2.0; Groom *et al.*, 2016 ▸) confirmed that the title compounds have not been previously published. Since hydrazides are very popular compounds and there are numerous entries in the database, the search was carried out for the specific fragment [OH—C—C(=O)—N—N—H], which represents the title structures albeit without the methyl substituent and excludes structures in which the terminal nitro­gen atom is engaged in a double bond. As a result of the search, six structures were found in which the defined fragment bears different substituents: JESVIN (Beckmann & Brooker, 2006 ▸); LACBOG (Andre *et al.*, 1993 ▸); RAVZIX and RAVZOD (Andre *et al.*, 1997 ▸); UVUTIQ (Noshiranzadeh *et al.*, 2017 ▸); VOJBUS (Abu-Safieh *et al.*, 2008 ▸); WETGEL (Chen *et al.*, 2021 ▸). Four of these structures (LACBOG, RAVZIK, RAVZOD, WETGEL) have a pyramidal nitro­gen, which is involved in the formation of inter­molecular hydrogen bonds similar to what is observed in the crystals of the title compounds.

## 5. Synthesis and crystallization

To a solution of 12.14 ml (0.3 mol) of methyl­hydrazine in 50 ml of 2-propanole were added dropwise 9.5 ml (0.1 mol) of ethyl glycolate at room temperature and the obtained solution was heated under reflux for 6 h. After completion of the reaction, the reaction mixture was evaporated under reduced pressure to remove excess of methyl hydrazine and the residual oil was dissolved in 25 ml of 2-propanole for crystallization to obtain (**1**) as white crystals. The filtrate was evaporated under reduced pressure and compound (**2**) was extracted using boiling benzene (5 × 30 ml). The precipitated solid from the combined benzene fractions was filtered off and recrystallized from 25 ml of ethyl acetate to obtain hydrazide (**2**) as white crystals.

**2-Hy­droxy-*****N*****’-methyl­acetohydrazide (1).** Yield 3.9 g (37.5%), m.p. 350–3551 K (2-propanole). ^1^H NMR (400 MHz, DMSO-*d*_6_) δ 9.16 (1H, *br.s*, NHNCO), 5.34 (1H, *br.s*, OH), 4.81 [1H, *br.s*, NH(CH_3_)], 3.82 (2H, *s*, CH_2_), 2.42 (3H, *s*, CH_3_). ^13^C NMR (101 MHz, DMSO-*d*_6_) δ 170.2, 61.0, 38.6. IR data (in KBr, cm^−1^): 3410, 3296, 2924, 1664, 1444, 1348, 1076, 880, 656, 572. MS (*m*/*z*, CI) 87.0 [*M* − OH]^+^,105.0 [*M* + H]^+^. Analysis calculated for C_3_H_8_N_2_O_2_: C, 34.61; H, 7.75; N, 26.91. Found: C, 34.67; H, 7.88; N, 26.90.

**2-Hy­droxy-*****N*****-methyl­acetohydrazide (2).** Yield 0.43 g (4.1%), m.p. 352–353 K (EtOAc). ^1^H NMR (400 MHz, DMSO-*d*_6_) δ 4.62 (2H, *s*, NH_2_), 4.17 (2H, *s*, CH_2_), 3.00 (3H, *s*, CH_3_). ^13^C NMR (101 MHz, DMSO-*d*_6_) δ 173.3, 59.8, 37.6. IR data (in KBr, cm^−1^): 3424, 3330, 2930, 1670, 1438, 1398, 1250, 1074, 1074, 808, 620, 572. MS (*m*/*z*, CI) 87.0 [*M* − OH]^+^, 105.0 [*M* + H]^+^. Analysis calculated for C_3_H_8_N_2_O_2_: C, 34.61; H, 7.75; N, 26.91. Found: C, 34.66; H, 7.80; N, 26.87.

## 6. Refinement

Crystal data, data collection and structure refinement details are summarized in Table 3 ▸. The low quality of the data is due to the fact that the quality of the crystals is not very good and we could not obtain bright distant reflections, which somewhat affects the final qu­anti­tative parameters·The O- and N-bound hydrogen atoms were identified in difference-Fourier maps and refined isotropically. The other H atoms were placed in calculated positions and refined using a riding model with *U*iso(H) = *nU*_eq_ of the parent atom (*n* = 1.5 for methyl groups and *n* = 1.2 for other hydrogen atoms).

## Supplementary Material

Crystal structure: contains datablock(s) 1, 2. DOI: 10.1107/S2056989024009526/yz2059sup1.cif

Structure factors: contains datablock(s) 1. DOI: 10.1107/S2056989024009526/yz20591sup2.hkl

Structure factors: contains datablock(s) 2. DOI: 10.1107/S2056989024009526/yz20592sup3.hkl

Supporting information file. DOI: 10.1107/S2056989024009526/yz20591sup4.cml

Supporting information file. DOI: 10.1107/S2056989024009526/yz20592sup5.cml

CCDC references: 2386931, 2386930

Additional supporting information:  crystallographic information; 3D view; checkCIF report

## Figures and Tables

**Table 1:** Hydrogen-bond geometry (Å, °) for **1**[Chem scheme1]

*D*—H⋯*A*	*D*—H	H⋯*A*	*D*⋯*A*	*D*—H⋯*A*
O1—H1⋯N2^i^	0.87 (5)	1.90 (5)	2.767 (5)	172 (4)
N1—H1*A*⋯O2^ii^	0.71 (4)	2.17 (4)	2.848 (5)	159 (4)

Symmetry codes: (i) 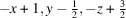; (ii) 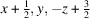.

**Table 2:** Hydrogen-bond geometry (Å, °) for **2**[Chem scheme1]

*D*—H⋯*A*	*D*—H	H⋯*A*	*D*⋯*A*	*D*—H⋯*A*
O1—H1⋯O2^i^	0.78 (4)	2.39 (4)	3.078 (4)	148 (3)
N2—H2*A*⋯O2^ii^	1.00 (3)	2.08 (3)	3.062 (4)	169 (2)
N2—H2*B*⋯O1^iii^	0.92 (3)	2.21 (3)	3.129 (4)	173 (2)

Symmetry codes: (i) 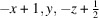; (ii) 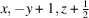; (iii) 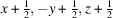.

**Table 3:** Experimental details

	**1**	**2**
Crystal data		
Chemical formula	C_3_H_8_N_2_O_2_	C_3_H_8_N_2_O_2_
*M*_r_	104.11	104.11
Crystal system, space group	Orthorhombic, *Pbca*	Monoclinic, *C*2/*c*
Temperature (K)	296	296
*a*, *b*, *c* (Å)	9.4484 (8), 7.0977 (7), 15.3781 (14)	11.646 (10), 9.304 (10), 10.514 (10)
α, β, γ (°)	90, 90, 90	90, 105.65 (4), 90
*V* (Å^3^)	1031.28 (16)	1097.0 (18)
*Z*	8	8
Radiation type	Mo *K*α	Mo *K*α
μ (mm^−1^)	0.11	0.11
Crystal size (mm)	0.3 × 0.2 × 0.1	0.2 × 0.15 × 0.09

Data collection		
Diffractometer	Bruker APEXII CCD	Bruker APEXII CCD
Absorption correction	Multi-scan (*SADABS*; Krause *et al.*, 2015 ▸)	Multi-scan (*SADABS*; Krause *et al.*, 2015 ▸)
*T*_min_, *T*_max_	0.602, 0.746	0.554, 0.746
No. of measured, independent and observed [*I* > 2σ(*I*)] reflections	10003, 909, 841	5355, 1259, 503
*R*_int_	0.072	0.099
(sin θ/λ)_max_ (Å^−1^)	0.595	0.650

Refinement		
*R*[*F*^2^ > 2σ(*F*^2^)], *wR*(*F*^2^), *S*	0.103, 0.199, 1.34	0.050, 0.126, 0.81
No. of reflections	909	1259
No. of parameters	77	77
H-atom treatment	H atoms treated by a mixture of independent and constrained refinement	H atoms treated by a mixture of independent and constrained refinement
Δρ_max_, Δρ_min_ (e Å^−3^)	0.32, −0.35	0.14, −0.16

Computer programs: *APEX2* and *SAINT* (Bruker, 2008 ▸), *SHELXT2018/2* (Sheldrick, 2015*a* ▸), *SHELXL2019/3* (Sheldrick, 2015*b* ▸) and *OLEX2* (Dolomanov *et al.*, 2009 ▸).

## supplementary crystallographic information

### 2-Hydroxy-N'-methylacetohydrazide (1) Crystal data

**Table:**

C_3_H_8_N_2_O_2_	*D*_x_ = 1.341 Mg m^−^^3^
*M**_r_* = 104.11	Mo *K*α radiation, λ = 0.71073 Å
Orthorhombic, *P**b**c**a*	Cell parameters from 3322 reflections
*a* = 9.4484 (8) Å	θ = 2.7–29.8°
*b* = 7.0977 (7) Å	µ = 0.11 mm^−^^1^
*c* = 15.3781 (14) Å	*T* = 296 K
*V* = 1031.28 (16) Å^3^	Block, colourless
*Z* = 8	0.3 × 0.2 × 0.1 mm
*F*(000) = 448	

### 2-Hydroxy-N'-methylacetohydrazide (1) Data collection

**Table:**

Bruker APEXII CCD diffractometer	841 reflections with *I* > 2σ(*I*)
Graphite monochromator	*R*_int_ = 0.072
φ and ω scans	θ_max_ = 25.0°, θ_min_ = 2.7°
Absorption correction: multi-scan (SADABS; Krause *et al.*, 2015)	*h* = −11→11
*T*_min_ = 0.602, *T*_max_ = 0.746	*k* = −8→8
10003 measured reflections	*l* = −17→18
909 independent reflections	

### 2-Hydroxy-N'-methylacetohydrazide (1) Refinement

**Table:**

Refinement on *F*^2^	Primary atom site location: iterative
Least-squares matrix: full	Hydrogen site location: mixed
*R*[*F*^2^ > 2σ(*F*^2^)] = 0.103	H atoms treated by a mixture of independent and constrained refinement
*wR*(*F*^2^) = 0.199	*w* = 1/[σ^2^(*F*_o_^2^) + (0.0435*P*)^2^ + 3.1357*P*] where *P* = (*F*_o_^2^ + 2*F*_c_^2^)/3
*S* = 1.34	(Δ/σ)_max_ < 0.001
909 reflections	Δρ_max_ = 0.32 e Å^−^^3^
77 parameters	Δρ_min_ = −0.35 e Å^−^^3^
0 restraints	

### 2-Hydroxy-N'-methylacetohydrazide (1) Special details

**Table:**

Geometry. All esds (except the esd in the dihedral angle between two l.s. planes) are estimated using the full covariance matrix. The cell esds are taken into account individually in the estimation of esds in distances, angles and torsion angles; correlations between esds in cell parameters are only used when they are defined by crystal symmetry. An approximate (isotropic) treatment of cell esds is used for estimating esds involving l.s. planes.

### 2-Hydroxy-N'-methylacetohydrazide (1) Fractional atomic coordinates and isotropic or equivalent isotropic displacement parameters (Å^2^)

**Table:**

	*x*	*y*	*z*	*U*_iso_*/*U*_eq_	
O1	0.5603 (3)	0.3926 (5)	0.8883 (2)	0.0278 (8)	
H1	0.575 (4)	0.275 (7)	0.875 (3)	0.022 (12)*	
O2	0.2107 (3)	0.4802 (6)	0.8076 (2)	0.0415 (10)	
N1	0.4200 (4)	0.4761 (5)	0.7415 (2)	0.0251 (9)	
N2	0.3656 (4)	0.5300 (5)	0.6594 (2)	0.0258 (9)	
H2	0.283 (5)	0.534 (7)	0.670 (3)	0.024 (13)*	
H1A	0.495 (5)	0.467 (5)	0.741 (3)	0.000 (10)*	
C1	0.4115 (4)	0.4115 (7)	0.8943 (3)	0.0317 (12)	
H1B	0.389895	0.509200	0.936369	0.038*	
H1C	0.372361	0.294298	0.916087	0.038*	
C2	0.3402 (4)	0.4592 (6)	0.8099 (3)	0.0251 (10)	
C3	0.3999 (5)	0.3911 (7)	0.5928 (3)	0.0371 (13)	
H3A	0.352088	0.422889	0.539704	0.056*	
H3B	0.370059	0.268531	0.611778	0.056*	
H3C	0.500296	0.390350	0.583003	0.056*	

### 2-Hydroxy-N'-methylacetohydrazide (1) Atomic displacement parameters (Å^2^)

**Table:**

	*U*^11^	*U*^22^	*U*^33^	*U*^12^	*U*^13^	*U*^23^
O1	0.0221 (16)	0.0291 (18)	0.0321 (19)	0.0036 (13)	−0.0061 (13)	−0.0040 (14)
O2	0.0129 (16)	0.070 (3)	0.042 (2)	0.0013 (16)	0.0002 (13)	0.0016 (19)
N1	0.0095 (19)	0.038 (2)	0.028 (2)	0.0033 (17)	0.0011 (17)	0.0077 (18)
N2	0.0155 (18)	0.035 (2)	0.027 (2)	0.0017 (17)	0.0006 (16)	0.0096 (17)
C1	0.024 (2)	0.046 (3)	0.025 (3)	0.000 (2)	0.0034 (19)	0.000 (2)
C2	0.022 (2)	0.022 (2)	0.031 (3)	−0.0022 (18)	0.0041 (19)	−0.0043 (19)
C3	0.035 (3)	0.048 (3)	0.028 (3)	−0.007 (2)	0.000 (2)	0.001 (2)

### 2-Hydroxy-N'-methylacetohydrazide (1) Geometric parameters (Å, º)

**Table:**

O1—H1	0.87 (5)	N2—C3	1.458 (6)
O1—C1	1.415 (5)	C1—H1B	0.9700
O2—C2	1.233 (5)	C1—H1C	0.9700
N1—N2	1.416 (5)	C1—C2	1.502 (6)
N1—H1A	0.71 (4)	C3—H3A	0.9600
N1—C2	1.300 (6)	C3—H3B	0.9600
N2—H2	0.80 (5)	C3—H3C	0.9600
			
C1—O1—H1	106 (3)	C2—C1—H1B	108.7
N2—N1—H1A	112 (3)	C2—C1—H1C	108.7
C2—N1—N2	122.4 (4)	O2—C2—N1	122.8 (4)
C2—N1—H1A	126 (3)	O2—C2—C1	119.8 (4)
N1—N2—H2	101 (3)	N1—C2—C1	117.4 (4)
N1—N2—C3	111.3 (4)	N2—C3—H3A	109.5
C3—N2—H2	112 (3)	N2—C3—H3B	109.5
O1—C1—H1B	108.7	N2—C3—H3C	109.5
O1—C1—H1C	108.7	H3A—C3—H3B	109.5
O1—C1—C2	114.3 (4)	H3A—C3—H3C	109.5
H1B—C1—H1C	107.6	H3B—C3—H3C	109.5
			
O1—C1—C2—O2	−179.6 (4)	N2—N1—C2—C1	−176.7 (4)
O1—C1—C2—N1	0.7 (6)	C2—N1—N2—C3	−124.1 (4)
N2—N1—C2—O2	3.5 (7)		

### 2-Hydroxy-N'-methylacetohydrazide (1) Hydrogen-bond geometry (Å, º)

**Table:**

*D*—H···*A*	*D*—H	H···*A*	*D*···*A*	*D*—H···*A*
O1—H1···N2^i^	0.87 (5)	1.90 (5)	2.767 (5)	172 (4)
N1—H1*A*···O2^ii^	0.71 (4)	2.17 (4)	2.848 (5)	159 (4)

Symmetry codes: (i) −*x*+1, *y*−1/2, −*z*+3/2; (ii) *x*+1/2, *y*, −*z*+3/2.

### 2-Hydroxy-N-methylacetohydrazide (2) Crystal data

**Table:**

C_3_H_8_N_2_O_2_	*F*(000) = 448
*M**_r_* = 104.11	*D*_x_ = 1.261 Mg m^−^^3^
Monoclinic, *C*2/*c*	Mo *K*α radiation, λ = 0.71073 Å
*a* = 11.646 (10) Å	Cell parameters from 1019 reflections
*b* = 9.304 (10) Å	θ = 2.8–30.1°
*c* = 10.514 (10) Å	µ = 0.11 mm^−^^1^
β = 105.65 (4)°	*T* = 296 K
*V* = 1097.0 (18) Å^3^	Block, colourless
*Z* = 8	0.2 × 0.15 × 0.09 mm

### 2-Hydroxy-N-methylacetohydrazide (2) Data collection

**Table:**

Bruker APEXII CCD diffractometer	503 reflections with *I* > 2σ(*I*)
Graphite monochromator	*R*_int_ = 0.099
φ and ω scans	θ_max_ = 27.5°, θ_min_ = 2.9°
Absorption correction: multi-scan (SADABS; Krause *et al.*, 2015)	*h* = −14→15
*T*_min_ = 0.554, *T*_max_ = 0.746	*k* = −12→12
5355 measured reflections	*l* = −13→13
1259 independent reflections	

### 2-Hydroxy-N-methylacetohydrazide (2) Refinement

**Table:**

Refinement on *F*^2^	Primary atom site location: dual
Least-squares matrix: full	Hydrogen site location: mixed
*R*[*F*^2^ > 2σ(*F*^2^)] = 0.050	H atoms treated by a mixture of independent and constrained refinement
*wR*(*F*^2^) = 0.126	*w* = 1/[σ^2^(*F*_o_^2^) + (0.060*P*)^2^] where *P* = (*F*_o_^2^ + 2*F*_c_^2^)/3
*S* = 0.81	(Δ/σ)_max_ < 0.001
1259 reflections	Δρ_max_ = 0.14 e Å^−^^3^
77 parameters	Δρ_min_ = −0.16 e Å^−^^3^
0 restraints	

### 2-Hydroxy-N-methylacetohydrazide (2) Special details

**Table:**

Geometry. All esds (except the esd in the dihedral angle between two l.s. planes) are estimated using the full covariance matrix. The cell esds are taken into account individually in the estimation of esds in distances, angles and torsion angles; correlations between esds in cell parameters are only used when they are defined by crystal symmetry. An approximate (isotropic) treatment of cell esds is used for estimating esds involving l.s. planes.

### 2-Hydroxy-N-methylacetohydrazide (2) Fractional atomic coordinates and isotropic or equivalent isotropic displacement parameters (Å^2^)

**Table:**

	*x*	*y*	*z*	*U*_iso_*/*U*_eq_	
O1	0.42646 (16)	0.2135 (2)	0.3887 (2)	0.0810 (7)	
H1	0.446 (3)	0.251 (4)	0.331 (3)	0.106 (14)*	
O2	0.61250 (14)	0.37066 (17)	0.37712 (16)	0.0690 (6)	
N1	0.68310 (16)	0.40430 (19)	0.59936 (19)	0.0505 (6)	
N2	0.6679 (2)	0.3683 (3)	0.7264 (2)	0.0644 (7)	
H2A	0.650 (2)	0.461 (4)	0.765 (3)	0.112 (11)*	
H2B	0.742 (2)	0.336 (3)	0.773 (3)	0.082 (9)*	
C1	0.50426 (19)	0.2560 (3)	0.5132 (2)	0.0603 (7)	
H1A	0.536650	0.171285	0.564015	0.072*	
H1B	0.459633	0.310151	0.562713	0.072*	
C2	0.60573 (19)	0.3481 (2)	0.4921 (2)	0.0480 (6)	
C3	0.7826 (2)	0.4983 (3)	0.5913 (2)	0.0684 (8)	
H3A	0.768514	0.593786	0.618254	0.103*	
H3B	0.788558	0.500277	0.502060	0.103*	
H3C	0.855570	0.462034	0.648489	0.103*	

### 2-Hydroxy-N-methylacetohydrazide (2) Atomic displacement parameters (Å^2^)

**Table:**

	*U*^11^	*U*^22^	*U*^33^	*U*^12^	*U*^13^	*U*^23^
O1	0.0648 (12)	0.1060 (18)	0.0656 (14)	−0.0291 (11)	0.0064 (11)	−0.0035 (12)
O2	0.0773 (13)	0.0799 (13)	0.0469 (11)	−0.0170 (9)	0.0118 (9)	0.0008 (9)
N1	0.0476 (11)	0.0557 (12)	0.0463 (12)	−0.0016 (10)	0.0095 (9)	0.0005 (10)
N2	0.0650 (16)	0.0812 (18)	0.0458 (14)	0.0090 (12)	0.0128 (12)	−0.0020 (12)
C1	0.0533 (14)	0.0660 (17)	0.0611 (17)	−0.0067 (13)	0.0146 (12)	−0.0046 (14)
C2	0.0486 (14)	0.0461 (15)	0.0464 (15)	0.0058 (11)	0.0079 (12)	0.0002 (12)
C3	0.0605 (16)	0.0652 (18)	0.0749 (19)	−0.0095 (13)	0.0102 (13)	−0.0013 (14)

### 2-Hydroxy-N-methylacetohydrazide (2) Geometric parameters (Å, º)

**Table:**

O1—H1	0.78 (4)	N2—H2B	0.92 (3)
O1—C1	1.432 (3)	C1—H1A	0.9700
O2—C2	1.251 (3)	C1—H1B	0.9700
N1—N2	1.434 (3)	C1—C2	1.523 (3)
N1—C2	1.345 (3)	C3—H3A	0.9600
N1—C3	1.472 (3)	C3—H3B	0.9600
N2—H2A	1.00 (3)	C3—H3C	0.9600
			
C1—O1—H1	110 (2)	C2—C1—H1A	109.6
N2—N1—C3	119.29 (19)	C2—C1—H1B	109.6
C2—N1—N2	117.8 (2)	O2—C2—N1	122.8 (2)
C2—N1—C3	122.9 (2)	O2—C2—C1	119.3 (2)
N1—N2—H2A	105.6 (16)	N1—C2—C1	117.9 (2)
N1—N2—H2B	104.1 (16)	N1—C3—H3A	109.5
H2A—N2—H2B	109 (2)	N1—C3—H3B	109.5
O1—C1—H1A	109.6	N1—C3—H3C	109.5
O1—C1—H1B	109.6	H3A—C3—H3B	109.5
O1—C1—C2	110.3 (2)	H3A—C3—H3C	109.5
H1A—C1—H1B	108.1	H3B—C3—H3C	109.5
			
O1—C1—C2—O2	−2.5 (3)	N2—N1—C2—C1	2.8 (3)
O1—C1—C2—N1	176.05 (19)	C3—N1—C2—O2	0.3 (3)
N2—N1—C2—O2	−178.7 (2)	C3—N1—C2—C1	−178.2 (2)

### 2-Hydroxy-N-methylacetohydrazide (2) Hydrogen-bond geometry (Å, º)

**Table:**

*D*—H···*A*	*D*—H	H···*A*	*D*···*A*	*D*—H···*A*
O1—H1···O2^i^	0.78 (4)	2.39 (4)	3.078 (4)	148 (3)
N2—H2*A*···O2^ii^	1.00 (3)	2.08 (3)	3.062 (4)	169 (2)
N2—H2*B*···O1^iii^	0.92 (3)	2.21 (3)	3.129 (4)	173 (2)

Symmetry codes: (i) −*x*+1, *y*, −*z*+1/2; (ii) *x*, −*y*+1, *z*+1/2; (iii) *x*+1/2, −*y*+1/2, *z*+1/2.
